# Identifying Unmet Needs in Major Depressive Disorder Using a Computer-Assisted Alternative to Conventional Thematic Analysis: Qualitative Interview Study With Psychiatrists

**DOI:** 10.2196/48894

**Published:** 2024-03-01

**Authors:** Michelle A Worthington, Richard H Christie, Aaron J Masino, Sarah M Kark

**Affiliations:** 1 AiCure New York, NY United States; 2 Department of Psychology Yale University New Haven, CT United States; 3 The School of Computing Clemson University Clemson, SC United States

**Keywords:** consumer health informatics, interview, major depressive disorder, medical informatics applications, needs assessment, psychiatry and psychology

## Abstract

**Background:**

The development of digital health tools that are clinically relevant requires a deep understanding of the unmet needs of stakeholders, such as clinicians and patients. One way to reveal unforeseen stakeholder needs is through qualitative research, including stakeholder interviews. However, conventional qualitative data analytical approaches are time-consuming and resource-intensive, rendering them untenable in many industry settings where digital tools are conceived of and developed. Thus, a more time-efficient process for identifying clinically relevant target needs for digital tool development is needed.

**Objective:**

The objective of this study was to address the need for an accessible, simple, and time-efficient alternative to conventional thematic analysis of qualitative research data through text analysis of semistructured interview transcripts. In addition, we sought to identify important themes across expert psychiatrist advisor interview transcripts to efficiently reveal areas for the development of digital tools that target unmet clinical needs.

**Methods:**

We conducted 10 (1-hour-long) semistructured interviews with US-based psychiatrists treating major depressive disorder. The interviews were conducted using an interview guide that comprised open-ended questions predesigned to (1) understand the clinicians’ experience of the care management process and (2) understand the clinicians’ perceptions of the patients’ experience of the care management process. We then implemented a hybrid analytical approach that combines computer-assisted text analyses with deductive analyses as an alternative to conventional qualitative thematic analysis to identify word combination frequencies, content categories, and broad themes characterizing unmet needs in the care management process.

**Results:**

Using this hybrid computer-assisted analytical approach, we were able to identify several key areas that are of interest to clinicians in the context of major depressive disorder and would be appropriate targets for digital tool development.

**Conclusions:**

A hybrid approach to qualitative research combining computer-assisted techniques with deductive techniques provides a time-efficient approach to identifying unmet needs, targets, and relevant themes to inform digital tool development. This can increase the likelihood that useful and practical tools are built and implemented to ultimately improve health outcomes for patients.

## Introduction

Digital health tools have the potential to advance health care efficiency, precision medicine, and patient health outcomes. Yet even the most high-performing digital tools using cutting-edge artificial intelligence and machine learning techniques are likely to be shelved if they do not address shared priorities among stakeholders [1] and their associated real-world unmet needs, leaving the promise of artificial intelligence and machine learning in health care underrealized [2]. There are currently 350,000 digital health apps worldwide that aim to address a range of functions (eg, condition management, wellness and prevention, and patient experience) and health conditions (eg, diabetes [3], neurological conditions [4], and psychiatric illnesses [5,6]). However, most of these apps are not regulated or clinically validated, and most are not widely used or integrated into clinical practice [7,8]. Although evidence suggests digital health tools are acceptable to both patients and clinicians, there are diverging needs, priorities, and attitudes among stakeholder groups within the digital tool ecosystem [1].

Gathering insights from key stakeholders is an essential step to ensure the development of digital tools that meet the needs of both patients and clinicians toward the goal of providing high-quality patient-centered care [9,10]. Using upstream stakeholder engagement methods can reveal actionable—but otherwise unforeseen—needs for targeted design and development of clinically impactful patient-facing digital tools with increased potential for widespread adoption [11,12]. Previous work has shown the benefits of qualitative research to assess hypothetical smartphone apps with content and function designs [13,14]. However, that work does not consider the motivation, or genuine need, for the app. Here we focus on the use of upstream qualitative research to first identify areas of real-world unmet need to serve as the foundation for hypothetical smartphone app ideation, design, and prototyping.

Qualitative research often involves designing and conducting a group of individual interviews and applying thematic analysis to transcripts of the resulting data. Conventional thematic analysis typically involves multiple researchers reviewing a subset of interview transcripts to identify themes [15]and developing a hierarchical system (codebook) of themes and subthemes (codes) to apply to sections of text (segments). The results of a conventional thematic analysis are typically presented in a table with broad summary themes, nested subthemes, and illustrative quotes from interview transcripts. The process of constructing the codebook can be time-consuming (eg, 3-5 times the amount of time taken to collect the data needed to review each interview [16]), subjective, and not always replicable [17,18].

While conventional thematical approaches are powerful for extensive investigations into a particular research area, a more time-efficient, scalable, and reproducible method is needed for digital health tool developers working in industry to swiftly identify key areas for the development of clinically meaningful tools. Indeed, in many settings of the digital health ecosystem, it is not feasible for researchers to dedicate substantial time to complete conventional thematic analysis. In some cases, it might be more appropriate to apply an automated text analysis that can be easily implemented by researchers, developers, and clinician scientists [19] to guide the identification of unmet needs and potential solutions. For example, recent work has applied text analysis to large qualitative research data sets to quickly identify common themes based on word frequency analysis [20-22]. Word combination frequency analysis provides a data-driven and repeatable approach to quickly identify frequently mentioned topics across a set of qualitative interview transcripts and potentially reduce the introduction of personal bias. Such approaches enable a faster and more convenient method to analyze a large amount of qualitative text data obtained from interviews with stakeholders [23]. Further, hybrid approaches that combine conventional thematic analysis with a data-driven inductive approach have the potential to leverage the strengths of both methods [24]. Importantly, such computer-assisted approaches to upstream qualitative research can be applied to engage and research any stakeholder group, including patients, to inform the development of clinically meaningful digital tools.

Given the limitations of conventional qualitative analysis—including the substantial time required for theme development—we demonstrate the utility of a hybrid approach leveraging the simplicity and accessibility of text analysis to identify stakeholder themes to support the initial stage of concept development for digital health tools. Specifically, we applied word combination analysis to a set of semistructured interviews with US-based psychiatrists specializing in treating outpatients with major depressive disorder (MDD) to reduce the amount of text for thematic analysis fivefold to facilitate uncovering common themes and unmet needs of clinicians and patients across advisors. We opted to demonstrate the use of simple text analysis over some of the more advanced natural language processing techniques to increase accessibility to those without advanced backgrounds in coding or computational techniques. We propose that this approach could serve as a straightforward and repeatable framework to identify unmet needs before concept development and implementation. We will discuss our findings around unmet needs in treating MDD and how this might affect digital tool development in psychiatry as an end-to-end demonstration of this method.

## Methods

### Ethical Considerations

The interviewed psychiatrists were originally recruited for market research purposes using the Guidepoint Expert Network (Guidepoint Global). The BRANY Institutional Review Board determined this study was exempt from review under category 4ii in 45 CFR 46.104(d). Participants were compensated for their time and participation. All reported data were stored in password-protected databases accessible only to approved study personnel.

### Recruitment and Data Collection

We used purposive criterion sampling to recruit 10 US-based psychiatrists specializing in treating MDD outpatients in a variety of practice settings, including academic medical centers and teaching hospitals, community-based mental health clinics, and private practices spanning urban and rural settings in the United States (Table 1). With only 2 exceptions, we recruited and interviewed psychiatrists who spent most of their time (≥50%) on direct MDD outpatient patient care and who had 10 or more years of experience postresidency. The interviewed psychiatrists were originally recruited for market research purposes using the Guidepoint Expert Network (Guidepoint Global).

**Table 1:** Stakeholder characteristics and treatment settings.

Stakeholder setting/US region			Clinic experience (years)		Direct patient care (% time)		Outpatient facing (% time)		MDD^a^ patient load (patients/week)
**Academic medical center or university teaching hospital**									
	Northeast	25		75		75		120	
	West	25		90		80		250	
	Southwest	20		30		99		20	
**Community hospital**									
	Midwest	25		95		50		35	
	Southwest	10		98		60		35	
	Southwest	7		100		50		50	
**Group or private practice**									
	West	28		95		95		35	
	Southwest	13		95		100		40	
	Mid-Atlantic	13		90		85		50	
	West	12		100		100		30	

^a^MDD: major depressive disorder.

Regarding sample size, previous work suggests most qualitative research data sets reach saturation—the point during data collection in which themes begin to repeat, new insights begin to wane, and future data collection becomes redundant—between 9 and 17 interviews [25]. Moreover, our inclusion criteria were designed to recruit a relatively homogenous sample of experienced clinicians specializing in outpatient MDD care, as we did not require any between-subject comparisons.

One researcher conducted individual, 1-hour, single-blinded, semistructured, recorded audio interviews following a predesigned interview guide (Multimedia Appendix 1). The interview guide was designed to include open-ended questions primarily targeted at understanding the clinicians’ experience in care management for MDD as well as the clinicians’ perceptions of patient experience in care management for MDD. We used MAXQDA Analytics Pro 2022 (version 22.1.1; VERBI GmbH) [26] to process and analyze the quality-checked interview transcripts (their official website provides help with using specific features). However, one can use other available software or open-source approaches to execute the simple word combination text analysis steps below.

### Data Preparation and Overview

All transcription text segments were auto-coded as either “interviewer” or “advisor,” depending on the speaker. Only “advisor” text was used during the text analysis stage.

**Figure 1:**
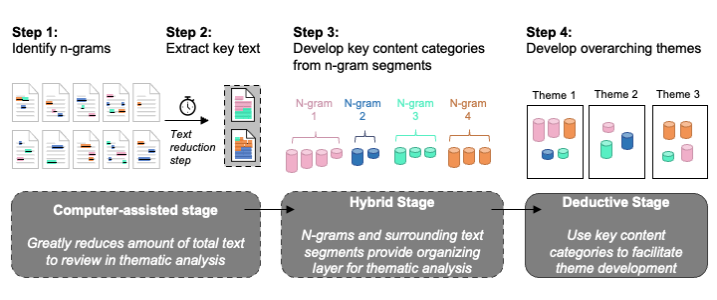
Visual depiction of the hybrid data-driven method to identify key themes and content categories. The dashed lines denote the 2 key differences between conventional thematic analysis and the present approach: a computerized text reduction step and a set of n-grams and their corresponding quotes that provide an organizing layer to assist with manual identification and the development of content categories.

The data-driven hybrid approach to identifying relevant themes involved a 4-step process (Figure 1): (1) During the computer-assisted stage, use MAXQDA (or other) software to identify the most frequent n-grams (n-word word combinations, n=2+ words) across the interview data set. (2) Extract the sentences containing each of those n-grams, along with the preceding and succeeding sentences relative to the sentence containing each n-gram. (3) During the “hybrid stage,” work iteratively with at least 1 other researcher to read and reread each computer-extracted text segment in order to inductively identify key content categories and assign a content category label to each text segment until 100% agreement is reached among researchers. Discordant content category assignments among researchers can be resolved during discussion. (4) Finally, in the “deductive stage,” manually examine the full list of key content categories to develop overarching themes and nest the key content categories under the broader themes characterizing priorities in the process of care management in treating MDD.

### Data Analysis

#### Identifying Word Combinations

First, we used the “word combination” feature in MAXQDA to identify the most frequently used phrases in the advisor’s responses. We used MAXQDA, but any software capable of word combination analysis can be used. We used n-word combinations instead of single-word frequencies to increase the specificity of theme identification. Within the search parameters, we required the resulting words to be at least 4 characters long. All words were lemmatized in English, and we applied the MAXQDA English language stop list [27] to remove articles, conjunctions, and other words likely to be redundant in the analysis. We additionally added word combinations that co-occurred with our disease state of interest to the stop list (eg, “major depression” and “mental health”), as these were unlikely to yield meaningful insights. Next, we filtered the resulting word combination frequencies to word combinations that also appeared in a majority (at least 60%) of the interviews. We opted for a majority threshold cut-off based on our sample (word combinations that appeared in at least 60% of interviews) to limit the subsequent research steps to a more focused set of word combinations that were not likely spoken just by random chance (ie, ≤50%). Our goal was to maximize the chance of developing a solution that would be impactful for the majority (>50%) of clinicians. However, depending on the goals, users may elect to use different thresholds. For completion, word combinations that appeared in 50% or more of the interviews are shown in the “Identifying Word Combinations” section, but only those that appeared in 60% or more of the interviews were included in the subsequent analysis.

Broadly, this first step served as a data-driven text analysis to identify commonly discussed themes or frequent expressions when discussing the care management process of the disease state of interest. Identifying key phrases used across stakeholders in a sample can give a sense of widely applicable needs and daily experiences. Identifying n-grams or word combinations is the crucial time-saving inductive step; however, to build meaning around the phrases, we recommend identifying key content areas by conducting follow-up deductive analyses of the context surrounding the word combinations.

#### Identifying Key Content Categories

Next, we extracted the resulting n-grams and their surrounding context for further analysis of the context in which these word combinations were uttered. Specifically, for each n-gram, we extracted the sentence containing the n-gram along with the 2 sentences before and after the appearance of each n-gram to provide context.

Next, 2 researchers systematically read the sentences surrounding each extracted word combination to understand the context in which the word combination was uttered. The researchers then worked iteratively through discussion and multiple independent readings to generate and assign relevant content categories for each n-gram. For expediency, the 2 researchers reconciled discordant labels through discussion until they reached 100% agreement. The researchers aimed to create content category labels for each word combination with definitions that were broad enough to accommodate multiple text segments but specific enough to distinguish among text segments from a given word combination. These researcher-generated content categories ultimately reflected the context surrounding the n-grams and provided a more in-depth understanding beyond the n-grams alone. Extracting and reviewing text around frequent and common n-grams helped to both focus the reading and deductive analysis of the text and substantially reduce the amount of text needed for review.

Next, we selected representative quotations for each content category to provide canonical examples of each of the chosen themes (Table S1 in Multimedia Appendix 2). This step represents a bridge between the text-analytic approach and the conventional deductive approach, in which researchers manually evaluate all the text to determine the appropriate content category represented in the segment. During this state, researchers review the text segments and apply their subject-matter expertise to sort the resulting text into subthemes. However, the text analytic step saves time and increases process transparency by examining only the segments in each interview where the n-gram appears. This should ultimately limit the scope of the analysis to pertinent segments constrained by the list of word combinations established in the first step.

Researchers may choose to stop at this level of analysis and proceed with the development of digital tool concepts if unmet needs and potential challenges are adequately identified. The word combinations and content categories may be substantial enough to provide sufficient context to understand how to proceed with digital tool development. Here, we further analyzed the word combinations and content categories in order to identify overarching themes, bringing the results of the hybrid method even closer to those yielded from conventional analysis.

#### Developing Overarching Themes

Once the content categories were identified as described in the previous step, key overarching themes emerged across the content categories and word combinations. At this stage, we also included word combinations that appeared in 50% of interviews to help guide the identification of more robust overarching themes that accommodated more word combinations. During reading and analysis of the resulting n-grams and their corresponding text segments, themes should start to emerge that may have been deduced using the more conventional analysis approach. In essence, the word combinations and key content categories established in the first and second steps are essentially transposed to develop these overarching summary themes, highlighting areas of need. In this approach, the word combinations are reorganized and presented with word combinations nested within broad summary themes. The goal of this final step is to summarize the findings from the first 2 steps into an alternative table structure that may assist in conceptualizing unmet needs for patient-facing digital tool development. By following the first 2 steps, theme identification in this final step becomes a much more efficient and transparent process as compared to a more conventional approach.

## Results

### Identifying Word Combinations

All word combinations appearing in 50% or more of the interviews are shown in Table 2. Only bigrams resulted from the analyses. There were no word combinations of ≥3 words. For a full summary of word combinations, content categories, detailed descriptions, and representative quotations, please refer to Table S1 in Multimedia Appendix 2.

**Table 2:** Frequently used word combinations listed by the percentage of interviews in which they were uttered and the total number of utterances across all interviews. The table elements are listed in descending order by the percentage of interviews in which they were uttered.

Word combination	Percentage of the interviews containing the word combination, %	Total utterances of the word combination across all interviews, n
Side effect	100	49
Make sure	100	24
Family history	70	12
Primary care	60	18
Substance abuse	60	9
Energy level	60	7
Really want	50	10
Treatment plan	50	9
Treatment resistant	50	9
Come back	50	8
Bipolar depression	50	7
Facial expression	50	7
Suicidal thought	50	7
Family member	50	6
Patient come	50	6
Feel comfortable	50	5
Good thing	50	5

Critically, after extracting the sentences containing the set of word combinations and their preceding and succeeding sentences, we observed that the total advisor text word count to manually review reduced to 21% of the original stakeholder text word count, demonstrating the use of the approach to assist with focusing theme extraction on key areas of text containing n-grams (ie, the highly repeatable text reduction step).

### Identifying Key Content Categories

After each researcher completed multiple readings of all word combinations and their surrounding contextual sentences, up to 5 content categories for each word combination were identified, resulting in a total of 14 unique content categories across phrases (Table S1 in Multimedia Appendix 2): administration, care management, common side effects, conceptualization, desired clinical information, differential diagnosis, medical comorbidities, medical history, medication monitoring and management, patient experience, risk assessment, risk for substance use, side effect monitoring, and treatment planning. The researchers aimed for ≤5 content categories for each word combination to balance specificity with generality. However, this target can be changed to accommodate larger or smaller studies that might require a higher or lower threshold to balance these goals. In total, there were 14 unique content categories, as some content category labels emerged under multiple word combinations (eg, care management emerged as a content category relevant to 2-word combinations, “side effects” and “primary care”).

### Developing Overarching Themes

In the final step, we identified overarching summary themes by transposing the word combinations and content categories identified in steps 1 and 2. In this analysis, we included word combinations uttered by 50% of advisors, which returned 11 additional word combinations (eg, “bipolar depression” and “facial expression”). The full results expanding on these overarching themes can be found in Table 3, with the word combinations listed as relevant word combinations that support content categories and overarching themes. Through this process, we identified four overarching summary themes: (1) evaluation, (2) medication decisions, (3) tracking symptom progression, and (4) factors contributing to treatment adherence.

**Table 3:** Overarching themes identified through transposing word combinations and content categories.

Overarching theme (with definition) and relevant key content categories			Relevant word combinations
**Evaluation: Providers expressed day-to-day challenges related to ruling out co-occurring psychiatric disorders (such as bipolar depression) and medical comorbidities and that they would benefit from more data about their patients.**			
	Differential diagnosis	“bipolar depression”, “make sure”, “substance use/abuse”	
	Medical comorbidities	“make sure”, “primary care”	
	Risk assessment	“suicidal thought”, “substance use/abuse”	
	Conceptualization	“suicidal thought”, “treatment resistant”, “facial expression”, “family history”, “family member”	
	Desired clinical information	“substance use/abuse”, “energy level”, “facial expression”	
**Medication decisions: Providers revealed their treatment planning choices are driven by leveraging known medication side effects to counteract patient symptom profiles (typically based on baseline energy levels).**			
	Medical history	“patient come”, “primary care”	
	Medication management and monitoring	“make sure”, “primary care”, “treatment resistant”	
	Treatment planning	“treatment plan”, “energy level”	
	Patient experience	“side effect”, “feel comfortable”	
	Common side effects	“side effect”	
**Tracking symptom progression: Providers were concerned about their lack of ability to track patient safety, triaging to a higher level of care, and co-occurring substance use outside of the clinic.**			
	Risk assessment	“suicidal thought”	
	Risk for substance [ab]use	“substance use/abuse”	
	Side effect monitoring	“side effect”, “energy level”	
**Factors contributing to treatment adherence: Providers highlighted the impact of adverse side effects on adherence and the critical role family members play in both patient adherence and providing them with symptom insights.**			
	Side effects	“side effect”	
	Patient experience	“feel comfortable”, “side effect”, “come back”	
	Care management	“family member”, “primary care”	
	Administrative	“come back”, “make sure”	

To create overarching themes, the researchers applied a similar process to the content category labels as they did to the word combinations—identifying a label and definition that would provide an appropriate umbrella term under which to nest multiple of the key content categories. To illustrate how we arrived at these broader themes, we will walk through the development of the “evaluation” theme. Analysis of the retrieved segments in the sentences surrounding the word combinations “bipolar depression,” “make sure,” and “substance [ab]use” converged on a content category best summarized as “differential diagnosis.” Through a similar process, segments surrounding the word combinations “suicidal thought,” “treatment resistant,” “facial expression,” “family history,” and “family member” converged on a content category best summarized as “conceptualization.” Ultimately, “differential diagnosis” and “conceptualization” fit together with “medical comorbidities,” “risk assessment,” and “desired clinical information” under the broad and overarching theme of “evaluation.”

## Discussion

### Overview

To ensure the development of clinically meaningful digital tools, developers must address real-world, unmet stakeholder needs. Understanding these needs at the outset of concept ideation and tool development ensures that developers are solving the most urgent challenges. Qualitative research is a powerful tool to understand the daily experiences of patients and clinicians and to better understand how potential digital tools will provide value and integrate seamlessly into a given environment. Here, we presented a hybrid data-driven approach to facilitate time-efficient discovery of unmet clinician needs to inform the direction of digital tool development in health care settings. With this approach, in the context of care management in MDD, we identified several key areas that are of interest to clinicians and would be appropriate targets for digital tool development.

### Using Key Content Categories to Inform Digital Tool Development

The key content categories are consistent with challenges identified in the literature, suggesting this approach can highlight real-world problems and point developers to further information in previously unknown areas of research literature. For example, the phrase “side effect” consisted of the following content categories: treatment planning, side effect monitoring, care management, patient experience, and common side effects. Further investigation into the literature encompassing this topic provides additional support for the selection of this area as a target of digital tool development. Burdensome treatment-related side effects are a leading cause of nonadherence and discontinuation of antidepressant medications [28] and have a negative impact on treatment outcomes [29]. Patients frequently experience side effects early in antidepressant treatment [30], but a lack of understanding of side effects [31] and barriers to communicating these side effects to clinicians, especially primary care providers [32], lead to early discontinuation and poor outcomes [29]. Some evidence exists that interventions addressing these early barriers to adherence, which include side effects, could improve adherence, communication between patient and provider, and, ultimately, treatment outcomes [33,34].

In the context of MDD care management, building digital tools that address patient and clinician concerns related to side effects, medication adherence due to side effects, and medication decisions based on side effects might best address one aspect of the current needs of clinicians as expressed through the interviews. The strengths of digital tools that could be leveraged in this context include facilitating side effect monitoring and reporting in between visits using smartphone capabilities, increasing patients’ understanding and expectations around side effects with on-demand psychoeducation through a mobile app or customized website, increasing clinicians’ awareness of the emergence of side effects through smartphone-based remote monitoring, or increasing and enhancing patient-provider communication through digital platforms.

Using a single topic as a guide for concept development may be a reasonable starting point for digital tool development; however, it is important to consider other related content categories that arise from this procedure. Incorporating other themes or content categories may bolster the initial concept and increase the likelihood that patients or clinicians will adopt a tool in clinical practice. In the context of MDD care management, the content category “administration” emerged as an important consideration for clinicians, signaling that workflow and administrative components of care management are also important to keep in mind while developing digital tools to ensure consideration of practical integration into workflows.

### Incorporating New Ideas With Existing Best Practices

Overall, the topics identified through the approach outlined in this study should line up with current thinking and best practices around digital tool development in health care. This includes, but is not limited to, prioritizing ethical considerations around data sharing and privacy [35,36], considering the legal implications of digital tool implementation [37], and considering the balance between addressing unmet needs and integrating tools into the current care management workflow [38]. While identifying specific themes and content categories for a given disease state will elucidate current unmet needs for clinicians, established guidelines around digital tool development should be included as well.

### Limitations

The approach outlined in this study has the potential to facilitate digital tool development across numerous clinical environments due to its ease of use and relative efficiency. Nevertheless, the comprehensive nature of conventional qualitative analysis may yield more nuanced findings from stakeholder interviews using a more deductive coding process. Moreover, limiting the amount of text for analytical review to those sentences surrounding frequent key word combinations across advisors is both a strength and a limitation of this method. While this approach increases efficiency, there could be a loss of sensitivity to important learnings outside of the extracted text segments, as well as some important one-off learnings uttered by only 1 advisor that could potentially be excluded from the text extracted for deductive analysis. Although the current sample size falls within a reasonable range to reach theme saturation across interviews [25], a more expanded sample size has the potential to reveal even more insights and accommodate analysis by psychiatry subspecialties (eg, addiction, child, adolescent, and geriatric psychiatrists). Furthermore, given that this method seeks to find consensus among spoken terms describing themes and unmet needs among clinicians and patients, it is possible that diverging opinions among stakeholders might be obscured. Finally, although the word combination analysis is 100% repeatable, further work would be needed to determine confirmability (ie, results confirmed by an independent set of researchers) [39].

### Future Directions

Here, we first interviewed clinicians because they are uniquely positioned within the digital health ecosystem to simultaneously identify and communicate patient needs, unmet clinician needs, potential for real-world clinical impact, and influence patient engagement with appropriate apps due to the trust patients place in their clinicians [1,7]. One challenge that blocks patient engagement with relevant digital tools is integration into clinician workflow [1], because clinicians have limited bandwidth to integrate patient-facing digital tools into their workflow. However, to ensure the most clinically relevant tools are created for and with patients, future work is needed to identify overlapping priorities between patients and clinicians. The method outlined here could be applied to patient interviews to reveal common and frequent unmet needs among patients.

The current framework provides a foundation for developers in the technology space to identify concepts that are worthy of further investigation and validation. Researchers following this framework may consider validating findings from this approach through follow-up methods such as: (1) triangulation studies (eg, test for convergence of results from this framework with focus group results or text from other sources, such as peer-reviewed articles about MDD), (2) quantitative survey methods, and (3) member-checking by presenting responses with the results to confirm the interpreted data and identified concept results resonate with their experience (respondent validation) [40] before moving further into conceptualization and prototyping.

### Conclusions

We presented a hybrid computer-assisted method for identifying unmet needs expressed in semistructured interviews, which provides an efficient and user-friendly approach to this problem. The presented method offers some of the efficiencies of a purely analytic approach (eg, topic modeling), while the incorporation of manual analysis of the surrounding context sentences offers some of the benefits of finding more interpretable and relevant concerns, as in a traditional qualitative analysis. By contrast, in topic modeling alone, researchers often consider the topics (lists of words) outside of their surrounding context when trying to interpret their meaning. Thus, this hybrid approach falls between these 2 approaches and gives digital health researchers a feasible approach for conducting upstream research to inform ideation and the development of high-impact patient-facing digital tools.

## Appendix

Multimedia Appendix 1 Semistructured interview guide.

Multimedia Appendix 2 Word combinations, key content categories, descriptions and representative quotations for word combinations appearing in at least 60% of all interviews.

## Abbreviations

MDD: major depressive disorder
