# Supplementary material for: Identifying Unmet Needs in Major Depressive Disorder Using a Computer-Assisted Alternative to Conventional Thematic Analysis: Qualitative Interview Study With Psychiatrists
Source: JMIR Form Res. 2024 Mar 1;8:e48894. doi: 10.2196/48894 (PMC10943432; doi:10.2196/48894)
Supplement: Multimedia Appendix 2 [file formative_v8i1e48894_app2.docx]

**Table S1.** Word combinations, key content categories, descriptions and representative quotations for word combinations appearing in at least 60% of all interviews. Note: The mention of any commercial drug names was replaced with “Drug X”.

| **Word combination** | **Key content category** | **Description** | **Example Interview Quote 1** | **Example Interview Quote 2** |
| --- | --- | --- | --- | --- |
| Side effect | Treatment planning | Use information about known side effects of antidepressants (sedation vs. activation) to inform treatment selection based on patient presentation (e.g., more slowed/sedated vs. more activated/insomnia). Consider switching or adding medications as side effects arise, depending on type and severity. | “This person can’t sleep, and they can’t eat, so I’m definitely going to use [Drug X] because it definitely has those side effects that we’re going to use to our advantage.” | “If they’re more anxious/agitated then something more calming or sedating. You know, so I can kind of use side effects to my advantage in some cases.” |
|  | Side effect monitoring | Pay close attention to emergence of side effects, especially in first phase of new medication trial. Monitoring may be more accessible with telehealth but more difficult without a full-body assessment. Track how emergence of side effects affects medication adherence. | “[We typically wait] to see how a patient may be doing, and at least for the initial few days, see if there’s any kind of initial side effects for that patient.” | “Their adherence may be more questionable and then they may be more willing to forgo it if they encounter any side effects versus somebody who may be more inclined to stay with it, despite a side effect.” |
|  | Care management | Improve patient care through patient collaboration, coordination with other health care providers and clear communication. Provide timely and accurate information about the pros and cons of treatment options, side effects, expected course of treatment, etc. | “Providing some education and reassurance that the side effects may go away, over time, whereas if it’s so significant that the patient wants to stop the medication, it’s important.” | “There’s a lot of uncertainty particularly if they’re coming into treatment for the first time. It could be overwhelming for them to take on a lot of new information and think about new medications and the chance that it may or may not work, and then their own fears about side effects or what these things may do to their brain so there’s all kinds of potential concerns that may come about.” |
|  | Patient experience | Take into consideration patient preferences about side effects in treatment planning. Consider timing of switching new medications depending on patients’ schedules (e.g., during a break in school). Address fears and concerns about possible side effects. | “The next medication change is going to take time and then there’s going to be a period of side effects and building up. I always tell patients it’s a good time to do it like over break when you’re not in the midst of stressful school or work.” | “They got really a lot of side effects, the common type of nausea, diarrhea, vomiting and then couldn’t eat, and so then they thought that they were dying from some stomach illness, and then they almost wanted to give up on psychiatric medications they just felt so bad and couldn’t remember how bad they felt being depressed. They were just focused on all the side effects.” |
|  | Common side effects | Listing and providing descriptions of different side effects, including weight gain, nausea, vomiting, diarrhea, dizziness, sleep, and sexual side effects. | “The patient’s symptoms may worsen during [the initial waiting period], maybe they’ll become suicidal, maybe they’ll become nauseated as a side effect of that particular agent that was chosen, maybe they’ll develop diarrhea, maybe they’ll become more irritable, maybe they’ll sleep too much, maybe they’ll be too hyperalert anything could happen.” | “The most common initially are gastrointestinal, so you may have nausea, vomiting, you could have dizziness, headache, you could have some patients get worse in terms of anxiety or insomnia and then longer-term weight gain and sexual side effects.” |
| Make sure | Differential diagnoses | Perform comprehensive assessment to rule out diagnoses that may complicate the clinical picture or treatment plan. If comorbidities exist, incorporate this into treatment planning. | “As part of the diagnosis, you want to make sure it’s not a bipolar spectrum condition. There are certain bipolar spectrum [disorders] that a lot of doctors might diagnose as not a bipolar type, as just unipolar depression.” | “They sometimes feel that they’re not able to perform as good as they used to and then some of them will want me to assess whether they have attention deficit disorder and so, one thing at a time, you really want to make sure this is a depressive disorder before you mix ADHD with that.” |
|  | Medical comorbidities | Conduct medical tests to rule out medical causes of psychiatric symptoms (e.g., thyroid problems, hormone imbalance, epilepsy). | “Medically, you want to make sure that this isn’t a medical condition mimicking depression. So, at baseline, we will often get a lab panel as well.” | “[What makes me feel confident in my treatment plan is] to make sure that they don’t have any comorbid medical conditions that would preclude the use of a particular medication and making sure they don’t have an allergy to it.” |
|  | Medication monitoring | Track effects of medications, especially after first starting a new medication, to ensure tolerability and alignment with patient’s priorities. | “I always have them come back within a week or two of starting a medication just to make sure it’s going well, that they’re not having side effects. If they don’t have a response at that time, I tell them that not having side effects is a win and probably takes more time.” | “There are pharmacies in my area that offer prepackaged medicines, we give out a pill minder boxes and nurses fill those boxes through home visits once a week. In other words, there are ways that we can try to help the patient to keep on course with taking medicines regularly. Can we make sure it happens 100% of the time? No, we can’t, but at least we do have some tools and resources that we can use to try to help.” |
|  | Risk assessment | Assess for levels of suicidal ideation, ensure patient safety throughout treatment process. | “Safety’s first and foremost, don’t get me wrong, and with all this I’m assessing for lethality and I’m making sure that they’re not an imminent risk for themselves for others, and if they are, then they need to get a higher level of care.” |  |
|  | Administration | Confirm patients have access to care through insurance coverage, appointment scheduling, and either transportation or telehealth accessibility. | “I wish I could magic wand all of that paperwork to be a lot more simple. Same with making sure about the verification of benefits--that’s a lot of back-and-forth process with my billing people.” | “One improved thing I would say with doing telepsychiatry is probably that the number of no-shows goes down. Patients are more readily able to connect in their home environment so it’s kind of a balance, I would say. Some of this information that may have been gleaned from observations of that patient, to making sure that we get that appointment done and completed, rather than the patient not show up at all.” |
| Family history | Conceptualization | Use information about psychiatric illness in first degree relatives to inform clinical conceptualization and treatment planning. Consider family history to rule in or out highly heritable disorders such as bipolar disorder or Schizophrenia. | “Tell me, medically, developmentally, psychosocial tell me about your family history and then let’s home in on symptoms and your history of psychiatric treatment.” | “We often may interview a family member, spouse, to get a sense if there’s anything we’ve missed with hypomania or that wasn’t reported and then we get family history, which is often helpful for bipolar I disorder in particular.” |
| Primary care | Medical history | Acquire relevant information about medical and treatment history from primary care provider or other referral source(s). | “You do want to get their medical history from the patient, if not from the primary care doctor. Usually, I get a summary of their current medical issues, if they have seen the primary care doctor, etc.” |  |
|  | Medication management | Use treatment history from primary care provider to inform medication decisions including switching, augmenting, starting, or stopping. | “In the US, my understanding is that two thirds of SSRI scripts are written by primary care physicians. So, these days, we get treatment naive patients, but the majority are referrals. They’re the patients who took one before—some PCPs will get adventurous and try a second medication, and then they’ll refer to us if the first or second treatment doesn’t work.” | “A lot of these patients if they came from primary care doctors, they are already on some sleep hypnotic or benzodiazepine too which is very addictive.” |
|  | Care management | Improve patient care through patient collaboration, coordination with other health care providers and clear communication. Provide timely and accurate information about the pros and cons of treatment options, side effects, expected course of treatment, etc. | “If the [osteoarthritis] is a big issue for them I’m going to send an electronic message, because I can do that in real time to their primary care provider and let them know what the patient told me about their pain level and ask what that’s already not happening can be done about it.” | “Conditions that commonly masquerade as depressive symptoms like thyroid, vitamin deficiencies, things like that, and we can do some labs, or I can coordinate care with your primary care doctor to get that together.” |
| Substance abuse/use | Risk for substance use | Determine risk factors for new or increased substance use during course of treatment including past substance use, increase in stressors, and factors related to presenting clinical problem (e.g., impulsivity). | “Agitated patients who are extremely anxious are very impulsive. There’s a risk you have to assess their comorbid substance abuse, so those patients, sometimes you do augment with atypical anti-psychotics.” | “With the lockdown, that was really bad on people’s mental health, a lot more substance abuse happened.” |
|  | Conceptualization | Assess how substance use fits into the clinical picture for presenting problem. Evaluate amount, duration, and type of use, and whether substance use causes or is caused by presenting problem. | “Major depressive disorder is my bread and butter, though. That and anxiety are often comorbid together. And also, substance abuse goes along with that too.” | “It’s so often these diagnoses are mixed up with substance use issues and personality issues, and ADHD and HIV. That’s how real people are.” |
|  | Desired clinical information | Types of information that would be helpful to have that clinicians don’t currently have access to. For example, more detailed information about behaviors and substance use in between sessions. | “They don’t tell us about substance use, so this tracker might be able to help me suggest if they are self-medicating with other things.” |  |
| Energy level | Treatment planning | Use information about known side effects of antidepressants (sedation vs. activation) to inform treatment selection based on patient energy levels (more slowed/sedated vs. more activated/insomnia, etc.). Consider how energy levels change over time with medication in choosing whether to switch or add medications. | “You have [Drug X], which might be more activating if they really sleep a lot, and they have no energy to get up. So, you want something to help them have some energy levels.” |  |
|  | Conceptualization | Use information about energy levels to inform clinical conceptualization and diagnosis. Consider high/low energy levels in the context of other presenting symptoms to arrive at appropriate diagnosis. | “I go through the DSM-5 criteria, ask about appetite, any suicidal thoughts, energy levels, concentration, any kind of guilty feelings. I get a sense of any kind of psychomotor retardation, which is also hard to see on video.” |  |
|  | Desired clinical information | Types of information that would be helpful to have that clinicians don’t currently have access to. For example, more detailed information about behaviors and substance use in between sessions. | “Number of steps taken, or speed of steps, or something like that to gauge energy levels...if I could somehow track that day to day or week to week, then we can have some objective data on how they are feeling.” |  |
